# Combating *Fusarium* Infection Using *Bacillus*-Based Antimicrobials

**DOI:** 10.3390/microorganisms5040075

**Published:** 2017-11-22

**Authors:** Noor Khan, Maskit Maymon, Ann M. Hirsch

**Affiliations:** 1 Department of Molecular, Cell and Developmental Biology, University of California, Los Angeles, CA 90095, USA; noor.612@gmail.com (N.K.); maskit@ucla.edu (M.M.); 2 The Molecular Biology Institute, University of California, Los Angeles, CA 90095, USA

**Keywords:** *Fusarium* sp., *Bacillus* sp., antagonism, antimicrobial peptide, biocontrol, plant protection

## Abstract

Despite efforts to control toxigenic *Fusarium* species, wilt and head-blight infections are destructive and economically damaging diseases that have global effects. The utilization of biological control agents in disease management programs has provided an effective, safe, and sustainable means to control *Fusarium*-induced plant diseases. Among the most widely used microbes for biocontrol agents are members of the genus *Bacillus*. These species influence plant and fungal pathogen interactions by a number of mechanisms such as competing for essential nutrients, antagonizing pathogens by producing fungitoxic metabolites, or inducing systemic resistance in plants. The multivariate interactions among plant-biocontrol agent-pathogen are the subject of this study, in which we survey the advances made regarding the research on the *Bacillus-Fusarium* interaction and focus on the principles and mechanisms of action among plant-growth promoting *Bacillus* species. In particular, we highlight their use in limiting and controlling *Fusarium* spread and infestations of economically important crops. This knowledge will be useful to define strategies for exploiting this group of beneficial bacteria for use as inoculants by themselves or in combination with other microbes for enhanced crop protection.

## 1. Introduction

Crop losses due to plant diseases pose a major threat to food security worldwide. The impact of losses ranges from a modest reduction of plant-growth measurements to more serious damage leading to plant death and reduced yield [1,2]. To prevent or control such pathogenic organisms and their infestations, many approaches have been undertaken, including the development of resistant varieties through plant breeding, the production of genetically modified resistant plants, and the use of chemical inputs such as fungicides. However, all have limitations. Development of resistant varieties requires time and moreover, resistance is not universal or permanent because the pathogen often evolves to overcome host plant resistance. Consequently, in the absence of effective and economically feasible alternatives, growers still rely heavily on easy-to-use conventional chemical pesticides and fungicides [3,4]. However, the utilization of these chemicals is strongly correlated with environmental contamination and disturbances in the natural balance of the soil microflora [4]. In addition, the presence of pesticide and fungicide residues on food may have adverse effects on human health, which has also raised significant concerns. Hence, with growing consumer awareness about low cost and sustainable agricultural methods, the need for effective biological control agents (BCA) is clear [5,6].

Among a variety of bacterial genera, species of *Bacillus*, *Pseudomonas*, and *Streptomyces* have been widely used as BCA [7,8]. However, plant-growth promoting (PGP) members of the genus *Bacillus* offer advantages over other microorganisms, owing to their ubiquity, ability to form endospores, and tolerance to fluctuating pH, temperature, and osmotic conditions [9], as well as their lack of pathogenicity [10,11,12]. *Bacillus* spp. colonize and establish robust interactions with roots by forming biofilms [13]. They promote plant growth by increasing nutrient uptake through siderophores and organic acids involved in P-solubilization, producing phytohormones, acting as bacterial antagonists, or inducing plant resistance against pathogens, as well as lysing fungal mycelia via hydrolytic enzyme synthesis and secretion [14].

Soil-borne phytopathogens are serious constraints to plant growth and productivity [15]. Most soil-borne pathogens can survive in the soil for *extended periods* of *time* where they remain dormant until they find a suitable host [16]. *Fusarium* species are globally important pathogens of agricultural plants and livestock, and also humans [17]. By inducing necrosis, wilting, and producing mycotoxins, *Fusarium* fungi are responsible for massive economic losses of many staple cereal food crops worldwide. In this review, we will focus on two of the most devastating species, *Fusarium oxysporum*, which causes vascular wilt, root rot, and damping-off in many plants [18] and also *F. graminearum*, which causes head blight on barley and wheat and infects many other cereal grasses as well [19].

Our aim is to highlight the sustainable strategies available for the control of *Fusarium* using beneficial *Bacillus* species and the mechanisms whereby they achieve disease control. We analyze the recent literature on the utilization of *Bacillus* species and their products in reducing crop damage by *Fusarium* species.

## 2. *F*. *oxysporum* and Vascular Wilt

The genus *Fusarium*, a well-known soil-borne plant pathogen, consists of a large number of plant-associated fungal species that have serious damaging effects on infected plants, such as eliciting chlorosis, necrosis, premature leaf drop, browning of the vascular system, and wilting, all of which subsequently cause significant yield losses. *Fusarium* species demonstrate a high level of host specificity, and, based on the plant species and cultivars they infect, are classified into more than 120 *formae speciales* and races [20]. Included in the genus are wilt pathogens such as *F. oxysporum*, *F. solani*, *F. graminearum*, and *F. verticillioides.*

One of the most economically destructive *Fusarium* species is *F. oxysporum* Schlecht. emend. Synd. et Hans, which infects more than 150 different plant hosts [21], including tomato (*Lycopersicon* spp.; *F. oxysporum* f. sp. *lycopersici*), banana (*Musa* spp.; *F. oxysporum* f. sp. *cubense*), cabbage (*Brassica* spp.; *F. oxysporum* f. sp. *conglutinans*), cotton (*Gossypium* spp.; *F. oxysporum* f. sp. *vasinfectum*), flax (*Linum* spp.; *F. oxysporum* f. sp *lini*), watermelon (*Citrullus* spp.; *F. oxysporum* f. sp. *niveum*), muskmelon (*Cucumis* spp.; *F. oxysporum* f. sp. *melonis*), onion (*Allium* spp.; *F. oxysporum* f. sp. *cepae*), pea (*Pisum* spp.; *F. oxysporum* f. sp. *pisi*), gladiolus (*Gladiolus* spp.; *F. oxysporum* f. sp. *gladioli*), and tulip (*Tulipa* spp.; *F. oxysporum* f. sp. *tulipae*) [20,22]. De Sain and Rep [23] reported that *F. oxysporum* secreted small, cysteine-rich proteins that contribute to its virulence. Additionally, the presence and absence of individual pathogenicity-related Secreted In Xylem (*SIX*) genes and sequence variation within the *SIX* genes can be used to discriminate between different *formae specialis* and races of *F. oxysporum* [24].

Recent reports of the close association between a polyphagous beetle and a new, but yet undescribed *Fusarium* species, have elicited major concern because this new interaction has resulted in an increase in avocado dieback disease in Los Angeles and Orange Counties, California [25]. The presence of *Fusarium*-induced dieback in urban landscapes throughout southern California is a potential threat to both industry and natural environments not only because of possible spread to commercial avocado fields, but also to native trees [26]. Mendel et al. [27] described a similar infestation that caused significant damage to commercial avocado orchards in Israel in 2009.

Pathogenic *F. oxysporum* isolates infect their hosts through the roots. They invade the xylem vessels and eventually result in lethal wilting of the infected plant. Wilting results from the restriction of movement of water in the vascular bundles [28], but the pathogenesis and invasion of plants by *F. oxysporum* in part is brought about by the toxic metabolites produced by the fungus.

In addition to eliciting major crop diseases, *F. oxysporum* in clinical settings causes systemic fusariosis in immunocompromised individuals [29]. López-Berges et al. [30] studied the velvet protein complex-based regulation of beauvericin mycotoxin production in *F. oxysporum* f. sp. *lycopersici* strain 4287. Of the four major components of the complex, deletion of *velA*, *velB*, and to a minor extent *velC*, caused distortions in the shape and size of microconidia, whereas *velA* and *laeA* were shown to be required for full virulence of *F. oxysporum* on tomato plants and immunodepressed mice. These data confirmed the critical contribution of the velvet protein complex in the expression of the gene cluster for beauvericin, a mycotoxin that functions as a virulence determinant.

## 3. *F. graminearum*, Killer of Cereals

*Fusarium graminearum* (teleomorph *Gibberella zeae* (Schwein.) Petch), the causative agent of *Fusarium* head blight (FHB) and crown rot (CR) on cereal crops is responsible for substantial economic losses every year [31]. *Fusarium* head blight is worldwide one of the most devastating fungal diseases affecting major cereal crops including wheat, barley, and maize [32,33]. The pathogen poses a two-fold threat: first, infested cereals show significantly compromised seed quality and yield, and second, the scabby grain is often contaminated with mycotoxins, which could cause serious human and livestock health damage [34,35].

## 4. Major *Fusarium* Disease Determinants: Mycotoxins

*Fusarium* fungi are widespread in the cereal-growing areas of the world and produce a range of mycotoxins, whose distributions are also varied [36]. Mycotoxins are secondary chemical metabolites synthesized by a variety of fungi. Numerous mycotoxins produced by *Fusarium* species with the ability to cause diseases in plants and animals have been described [37].

Although fusaria are found in all cereal-growing regions, they exhibit significant geographical differences in their natural distribution, and so do their corresponding mycotoxins, the levels of which are influenced by a number of factors with environmental conditions, crop production, and storage methods being the major determinants [38]. Toxins produced by *F. oxysporum* include fusaric acid, beauvericin (BEA), moniliformin, naphthazarins, and sambutoxin [39]. Important mycotoxins produced by other *Fusarium* species that are hazardous to human and animal health include fumonisins, the trichothecenes (T2-toxin, nivalenol, and deoxynivalenol) and zearalenone [40]. Hernandes et al. [41] demonstrated that filtered *Fusarium oxysporum* extract induced an inflammatory reaction and programmed cell death in rat skin. Similarly, de Melo and Piccinin [42] reported the toxic activity of *F. oxysporum* where the fungal culture extracts provoked reactions that produced withering in cucumber cells and plantlets, leading to cell death.

Strains of the *F. graminearum* species complex (FGSC) cause head blight and spike disease, which is of significant economic importance. The reduced grain quality comes about from the accumulation of a diversity of *F. graminearum* mycotoxins. This pathogen typically produces one of the three potential trichothecene profiles: (i) deoxynivalenol (DON) and 3-acetyldeoxynivalenol (the 3ADON chemotype); (ii) DON and 15-acetyldeoxynivalenol (the 15ADON chemotype); or (iii) nivalenol (NIV), its acetylated derivatives, and low levels of DON (the NIV chemotype) [43]. Deoxynivalenol (DON), also known as vomitoxin, is the most frequently detected trichothecene and contaminant in grain samples. It causes multiple effects on eukaryotic cells with inhibition of protein synthesis being the primary one [44,45]. Maier et al. [46] reported that the mycotoxin DON was the major infection causing agent for *F. graminearum* disease in wheat spikes.

## 5. Management of *Fusarium* Wilt

*Fusarium* wilt has been a problem for many years and numerous strategies have been proposed to control this fungal pathogen [47]. However, attempts to control the disease have shown limited success, mainly due to the emergence of new pathogenic races [48]. The documented methods employed for controlling wilt infections are: cultural, biological, i.e., resistance development, and chemical such as the use of fungicides [49] and/or natural products [50]. Control of *Fusarium* infections is usually accomplished by applying benomyl, prochloraz, carbendazim, fludioxonil, bromuconazole, or azoxystrobin [51]. Everts et al. [52] tested the efficacy of three soil-applied fungicides, prothioconazole, acibenzolar-S-methyl, and thiophanate-methyl, each of which reduced *Fusarium* wilt of field-grown watermelon. Nevertheless, the best control option for *Fusarium* wilt disease, when available, is using resistant cultivars. *Fusarium* wilts are difficult to manage without incorporating durably resistant cultivars. A number of other options that can help reduce the severity of the disease exist, but they are not always effective by themselves. They include: soil fumigation with 1,3-dichloropropene + chloropicrin [53], chloropicrin [54], methyl isothiocyanate [54,55], propylene oxide [56], and sodium azide [56]. Other strategies used are the avoidance of infected fields, cover cropping [57], crop rotation, and the use of other agro-chemicals.

The difficulties in controlling *Fusarium* wilt have stimulated renewed interest in biological control and the use of beneficial plant growth-promoting bacteria (PGPB) as a disease management alternative. For this purpose, plant root-colonizing, beneficial bacteria and fungi including species of *Pseudomonas (Pseudomonas fluorescens*, *P. putida)* [58], *Bacillus (Bacillus subtilis*, *B. polymyxa*, *and B. amyloliquefaciens)* [59], non-pathogenic *Fusarium* [60], and Actinobacteria [61] have been selected. However, *Bacillus* species are preferred not only for their ability to form stress-resistant endospores, but also for their safety in handling.

Another approach to improve the reliability and level of performance is to combine biocontrol agents in strain mixtures [62]. Lutz et al. [63] proposed using mixtures of antagonistic bacteria and fungi, and this approach has proven to be more effective than single strain treatments against a variety of plant diseases. Dunlap et al. [64] found that mixing biocontrol *Bacillus subtilis* OH 131.1 with *Cryptococcus flavescens* (telomorph: *Filobasidiella*) led to more effective control of *Fusarium* head blight infection in wheat under both greenhouse and field settings. A study by Zalila-Kolsi et al. [65] reported the use of a tripartite combination of *B. amyloliquefaciens*, *B. subtilis*, and *Paenibacillus polymyxa* that led to the highest protection rate of wheat against *F. graminearum*, when compared to the strains used individually. This result indicates that combining compatible BCAs could be a strategic approach in controlling plant diseases.

For developing a successful plant disease management program, examination of the sum total of interactions that occur between plant and pathogen, and the subsequent elimination of those interactions, or favoring those that tip the balance in favor of the plant is essential. Reduction of pathogen viability, i.e., population density, and/or functionality, and the ability to infect the host effectively are the keys to a successful antagonist. Fruitful management of *Fusarium* wilt diseases of vegetable crops needs to be multi-faceted and should include such strategies as breeding or introducing genes for host resistance, growth of cover crops that improve soil organic matter, enhancement of plant nutrition, and avoidance of diseased transplants.

## 6. Biocontrol Attributes of Various *Bacillus* Species

Beneficial bacteria (particularly those belonging to *Bacillus* and the closely related genus *Paenibacillus*) that reside in close association with plant roots are of particular interest for their antifungal and plant protective properties [66]. Some *Bacillus* spp. directly antagonize fungal pathogens by competing for niches and essential nutrients [67], or by producing fungitoxic compounds [68], and also by inducing systemic acquired resistance [69]. Siderophore production by bacteria is another attribute that promotes plant growth in two ways: (1) by supplying iron to plants; and (2) depriving the fungal pathogens of this essential nutrient. Heidarzadeh and Baghaee-Ravari [70] reported that a siderophore-producing *B. pumilis* strain was an effective BCA for *Fusarium* wilt of tomato. Production of extracellular enzymes by biocontrol bacteria that causes lysis of the phytopathogenic fungal cell wall is a well-documented phenomenon [71]. DasGupta et al. [72] performed scanning electron microscopic studies that demonstrated alteration and distortion in the hyphal cell walls of *F. oxysporum* f. sp. *ciceri* in response to chitinase and β-1,3-glucanase produced by *Paenibacillus lentimorbus* B30488. In vitro studies showed that chitinase produced by *B. subtilis* caused lysis of the postharvest yam pathogen *F. oxysporum* [73]. This result was further validated by in vivo experiments where *B. subtilis* application inhibited the incidence *of F. oxysporum* by 83% in wound cavities of yam tubers. Moreover, Zhao et al. [74] found that *B. subtilis* strain SG6 exhibited strong antagonism against *F. graminearum* in dual culture plate assays and inhibited sporulation in the pathogen. These studies were further complemented by SEM and TEM analyses that revealed evidence of pathogen cell wall lysis by the biocontrol strain. Spectrometric analysis of the bacterial culture supernatant showed that the antimicrobial peptides (AMP), fengycin and surfactin, were present.

Recently, Veliz et al. [4] reviewed the literature on the importance of chitinases in pathogen control and the use of chitinolytic microorganisms as an effective solution in controlling fungal diseases. Gomaa [75] showed the efficacy of seed treatment of chitinase purified from *Bacillus thuringiensis* NM101-19 in controlling *Fusarium* infection in soybean. Studies report some other mechanisms employed for biocontrol. Yuan et al. [76] reported that *Bacillus amyloliquefaciens* NJN-6 produces numerous volatile compounds (VOCs) that restrict growth and spore germination of *F. oxysporum* f. sp. *cubense*. *Bacillus fortis* IAGS162 earlier had been shown to induce systemic resistance in tomato plants against *Fusarium* wilt disease [77]. Additional findings by Akram et al. [78] identified phenyl acetic acid (PAA) produced by *B. fortis* IAGS162 as the major factor responsible for efficient bacterial colonization in the plant rhizosphere and the subsequent suppression of *Fusarium* wilt disease. Recently, *B. simplex*, an emerging PGPB, has been shown to inhibit the growth of three different *Fusarium* strains. This strain and a newly identified *B. subtilis* strain also promoted legume plant growth especially when coinoculated with *Rhizobium* [79].

### 6.1. Bacillus Peptide Antibiotics

Several species of genus *Bacillus* are known to produce antibiotics, of which the peptide antibiotics form a dominant class. Based on their biosynthetic pathway, these metabolites can be grouped into two main categories, the ribosomally synthesized peptides (including bacteriocins) and small peptides synthesized enzymatically by non-ribosomal pathways [80].

**Figure 1:**
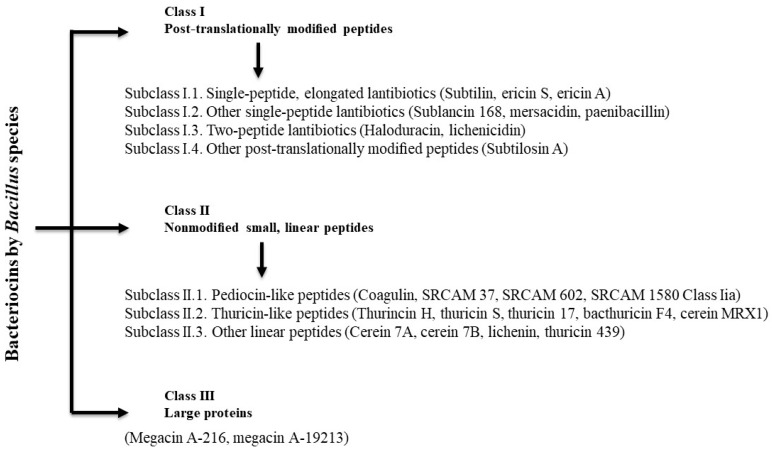
Classification of bacteriocins produced by *Bacillus* species (based on Abriouel et al. [81]).

Lanthipeptides (Class I) are a group of post-translationally modified peptides characterized by the presence of lanthionine (Lan) or methyllanthionine (MeLan) bridges. Currently, they are classified into four subclasses, but only gene clusters of two of the subclasses of lanthipeptides have been identified in *Bacillus* spp. strains [81,82]. On the basis of classification provided by Abriouel et al. [81], bacteriocins can be classified into post-translationally modified and non-modified peptides (Class II; also divided into subclasses) as summarized in Figure 1. Large peptides, such as the megacins (derived from *B. megaterium*), make up Class III.

### 6.2. Non-Ribosomal Biosynthesized Peptides

**Figure 2:**
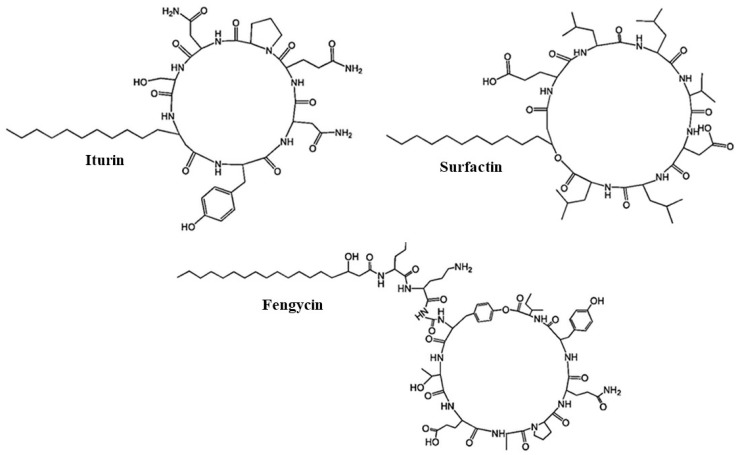
The structures of iturin, surfactin, and fengycin; all share a common structure consisting of a lipid tail linked to a short cyclic peptide. The derivatives of compounds in each group come from different amino acid components [85].

The non-ribosomal synthesis of peptide antibiotics takes place through a multistep mechanism that includes the selection and condensation of amino acid residues such as cyclic lipopeptides (iturin group) and macrolactones (surfactins, fengycins, and plipastatins) [83]. Large multienzymes known as Non-Ribosomal Peptide Synthetases (NRPS), which are composed of modularly arranged catalytic domains [84], catalyze their biosynthesis. Structural representations of non-ribosomally synthesized peptide antibiotics are illustrated in Figure 2.

In the context of biocontrol of wilt diseases, the three families of *Bacillus* lipopeptides, surfactins, iturins, and fengycins, have been extensively studied for their antagonistic activity [68]. Recently, Geissler et al. [86] established a high-performance thin-layer chromatography (HPTLC) method for the identification and simultaneous quantification of the cyclic lipopeptides surfactin, iturin A, and fengycin, in *Bacillus* culture samples. Sandrin et al. [87] reported strong antifungal activity of iturins and fengycins against fungal pathogens, whereas surfactins were not found to be very toxic by themselves. Nevertheless, they promoted the antagonistic potential of iturin A [88]. Surfactins have been suggested to assist in the formation of stable biofilms on host surfaces, thereby protecting the beneficial bacteria from antibiosis and competition exerted by other microorganisms [89]. Vitullo et al. [90] demonstrated the antifungal activity of purified surfactin from *B. amyloliquefaciens*, which suggested an important role of this molecule in the biocontrol of *F. oxysporum*. *Bacillus amyloliquefaciens* S76-3 isolated from diseased wheat spikes has strong antagonistic activity against *F. graminearum* [91]. Reverse-phase high performance liquid chromatography and electrospray ionization mass spectrometry analyses revealed that strain S76-3 produces three classes of cyclic lipopeptides, including iturin, plipastatin, and surfactin. However, only the iturins and plipastatin were responsible for biocontrol effectiveness.

Blacutt et al. [92] reported the presence of fengycin and surfactin lipopeptides in culture supernatants of *B. mojavensis* RRC101 that inhibited the growth of *F. verticillioides*. Microscopic analysis revealed hyphal distortions, vacuolization, and lysis of *F. verticillioides* on exposure to fengycin. Li et al. [93] reported that the encounter between *B. amyloliquefaciens* SQR9 and *F. oxysporum* resulted in an increased production of bacillomycin and fengycin, whereas when exposed to *Rhizoctonia solani* and *F. solani*, the production of surfactin increased in *B. amyloliquefaciens* SQR9, but fengycin production decreased. Zihalirwa Kulimushi et al. [94] observed much higher iturin and fengycin production in *B. subtilis* 98S on co-culturing it with *Pythium* and *Fusarium*, but a similar observation was not recorded in the presence of *Botrytis* [68]. Thus, it appears that activation of different suites of lipopeptides depends on the interacting fungal challenger and is likely to be strain-specific.

The presence of AMP biosynthetic genes has been linked to the antagonism of plant pathogens in several *Bacillus* strains, particularly the genes *ituC*, *bmyB*, *fenD* and *srfAB* [95]. The simultaneous production of different AMPs is important for an effective control of plant diseases and also is a key factor determining the broad range of antagonistic activity in *Bacillus* species. The dominance of these genes in *Bacillus* strains associated with plants strengthens the competitive role of surfactin, iturin, bacillomycin, fengycin and bacilysin in the improving the fitness of strains in fluctuating environmental conditions. The use of AMP gene markers may assist in the selection of putative BCA of plant pathogens [96].

Additional recent investigations have shed light on the fact that these lipopeptides can also influence the ecological fitness of the producing strain in terms of root colonization and their long-term persistence in the rhizosphere [94]. They also play a key role in the beneficial interaction of *Bacillus* species with plants by stimulating host defence mechanisms [97]. Choudhary and Johri [98] summarized various aspects of research on *Bacillus* plant growth-promoting rhizobacteria (PGPR) eliciting ISR, which leads to significant reductions in plant diseases coupled with enhancement in overall plant health.

Degradation of pathogen’s virulence factors by biocontrol bacteria is another promising strategy for controlling pathogen proliferation and subsequent disease infestations. For example, Guanhua et al. [99] studied the capability of *Bacillus licheniformis* CK1 on degrading zearalenone, thereby reducing its adverse effect on post-weaning female piglets.

## 7. Conclusions

**Figure 3:**
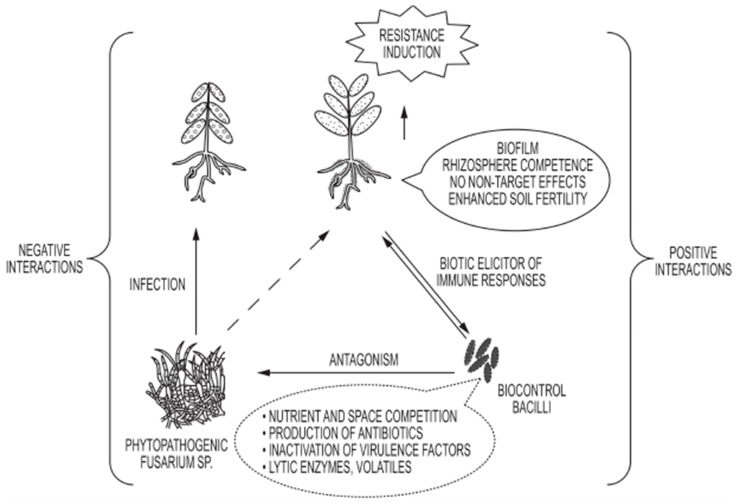
Schematic diagram illustrating the dynamic multifactorial interaction between *Bacillus* and *Fusarium* spp., and their relative impact on plant health.

During the past decades, chemical fungicides have been the main strategy to manage *Fusarium* infections. However, because of their non-targeted and negative effects on humans and the environment, beneficial bacteria are increasingly being tested as substitutes for the environmentally damaging chemicals. Beneficial strains of *Bacillus* rank high for their potential as BCA in part, not only for their PGPR traits, but also because they are spore-forming bacteria, which makes them easy to formulate and preserve as inoculants. With their ability to produce a range of metabolites that stimulate plant growth and reduce pathogen attack, either by suppressing fungal growth or inducing the plant immune system against pathogens, members of *Bacillus* and allied genera are preferred over other types of BCA. An overview of the multivariate influence of *Bacillus* on the interaction of pathogenic *Fusarium* and plant health is illustrated in Figure 3.

This literature survey highlights the need for a cost-effective, commercial *Bacillus*-based biofungicide that is effective against the major *Fusarium* species that cause disease. We have drawn attention to some of the key features of the biocontrol aspects of several *Bacillus* beneficial strains and have focused on the two major *Fusarium* pathogens. However, numerous *Bacillus* species that may be used as BCA and as biofertilizers are still being discovered and so far, remain untested. More research into the diversity of *Bacillus* strains and their mechanisms of biocontrol is needed to achieve an understanding of the interactions of these bacteria, particularly with other beneficial microbial inoculants.

Finally, consortia of microbial populations are more likely to harness more benefits in terms of reducing plant disease, improving crop growth, and maintaining environmental health through sustainable agriculture. More studies need to be pursued to test this hypothesis.
